# Effectiveness of galvanic vestibular evoked myogenic potential for evaluation of Meniere’s disease

**DOI:** 10.1016/j.bjorl.2021.11.010

**Published:** 2022-02-21

**Authors:** Ying Cheng, Yuzhong Zhang, Zichen Chen, Weijun Ma, Yanfei Chen, Qing Zhang, Min Xu

**Affiliations:** a Department of Otolaryngology, Head and Neck Surgery, The Second Affiliated Hospital of Xi’an Jiaotong University, China; b Xinhua Hospital of Shanghai Jiao Tong University School of Medicine, China

**Keywords:** MD, Meniere’s disease, cVEMP, cervical vestibular-evoked myogenic potential, oVEMP, ocular vestibular-evoked myogenic potential, ACS, Air-Conducted Sound, GVS, Galvanic Vestibular Stimulation, Meniere’s disease, Cervical vestibular-evoked myogenic potential, Ocular vestibular-evoked myogenic potential, Air-Conducted Sound, Galvanic Vestibular Stimulation

## Abstract

**Objectives:**

Although the cause of Meniere’s disease (MD) is not fully understood, endolymphatic hydrops is widely believed to be responsible for MD. Previous studies have used Air-Conducted Sound (ACS)-induced Vestibular Evoked Myogenic Potentials (VEMPs) to evaluate otolithic function in patients with MD. However, the use of Galvanic Vestibular Stimulation-VEMPs (GVS-VEMPs) with other vestibular tests in MD has been rare. This study aimed to explore the application of galvanic VEMPs in assessing MD.

**Methods:**

Normal individuals and patients with unilateral definite MD were included in this retrospective study. All participants underwent pure tone audiometry. Ocular and cervical VEMPs induced by GVS, and ACS were recorded. The characteristic parameters of VEMPs (n1 latency, p1 latency, amplitude, and AR) were analyzed.

**Results:**

The provocation rates of GVS-VEMPs did not differ between MD patients and control individuals. Compared with ACS, GVS could evoke potentials with longer latencies. MD patients presented GVS-VEMPs with lower amplitudes and ACS-cVEMP with shorter latencies and had a higher response rate in GVS-oVEMP. However, no differences or correlations were found in the characteristic parameters of GVS-VEMPs among the different stages of MD.

**Conclusions:**

GVS is as effective as ACS for inducing VEMP, and GVS-VEMP recording can detect retrolabyrinthine degeneration in MD. Further research is needed to assess the utility of GVS-VEMP in the evaluation of MD severity.

**Level of evidence:**

Level 4.

## Highlights

• GVS and ACS-VEMPs were compared in patients with unilateral MD and normal controls.
• GVS was as effective as ACS for inducing VEMPs, and even faster than ACS.
• GVS-VEMP recording can detect retrolabyrinthine degeneration in MD.

## Introduction

Meniere’s disease (MD) is a disease of the inner ear with characteristic manifestations of vertigo, tinnitus, hearing loss, and a pressure felt inside the ear.^1^ Although the cause of MD is not fully understood, endolymphatic hydrops is widely believed to be responsible for MD. A variety of potential conditions result in over-production or inadequate resorption of endolymph in the inner ear,^2^ which leads to distension of the endolymphatic spaces in the inner ear and gives rise to the above symptoms.

In clinical practice, the caloric test, video Head Impulse Test (vHIT), and Vestibular-Evoked Myogenic Potentials (VEMPs) have been commonly used to evaluate vestibular function in cases of MD. During the caloric test, cold or warm water is irrigated into the external auditory canal to assess the function of the horizontal semicircular canal. The vHIT can detect dysfunction in individual vertical, horizontal, or posterior semicircular canals. There are two frequently used types of VEMPs: cervical VEMPs (cVEMPs) and ocular VEMPs (oVEMPs). cVEMPs are recorded from the Sternocleidomastoid Muscles (SCMs) and reflect the function of the saccule and its afferent pathway,^3^, ^4^ whereas oVEMPs are recorded from the inferior oblique muscle and reflect the function of the utricle and its afferent pathway.^5^, ^6^ VEMPs can be evoked by Air-Conducted Sound (ACS), Bone-Conducted Vibration (BCV), and Galvanic Vestibular Stimulation (GVS).^7^, ^8^, ^9^ In general, ACS is thought to trigger VEMPs mainly at the receptor level, while GVS directly triggers the vestibular afferents. Therefore, GVS-triggered VEMP (GVS-VEMP) can be used for the differential diagnosis of labyrinthine versus retro-labyrinthine lesions.

Previous studies have used ACS-induced VEMP to evaluate otolithic function in patients with MD.^10^, ^11^ We hypothesized that GVS-VEMP may offer additional information regarding the localization and severity of MD. The present study was designed to test this hypothesis by comparing the VEMPs evoked by GVS and ACS in patients with unilateral MD as well as normal controls.

## Methods

### Study participants

Patients diagnosed with unilateral MD in the Otorhinolaryngology Head and Neck Surgery Department of the Second Affiliated Hospital of Xi’an Jiaotong University from August 2018 to September 2019 were enrolled in our study. All patients were diagnosed using the criteria for Menière’s disease jointly formulated by the Classification Committee of the Bárány Society, The Japan Society for Equilibrium Research, the European Academy of Otology and Neurotology (EAONO), the Equilibrium Committee of the American Academy of Otolaryngology–Head and Neck Surgery (AAO–HNS) and the Korean Balance Society in 2015.^12^

Age- and gender-matched normal individuals were recruited as a control group. We obtained an accurate medical history and performed a careful physical examination for all participants. They all underwent audiologic tests (i.e., pure tone audiometry, tympanometry, distortion product otoacoustic emission, auditory brainstem response, electrocochleogram), otoscopy, temporal bone Computed Tomography (CT) examination, and VEMP testing. Individuals with any problem in the external or middle ear, eyes, or cervical spine were excluded. Informed consent was obtained from all study participants. The present study was approved by the Ethics Committee of Second Affiliated Hospital of Xi’an Jiaotong University (2016205).

### VEMP testing

VEMP testing was performed using an electromyographic evoked potential system (MEB-9404C, NIHON KOHDEN, Japan). For cVEMP recording, three electrodes were placed as follows: an active electrode on the middle of Sternocleidomastoid (SCM), a reference electrode on the lateral end of the upper sternum, and a ground electrode on the forehead. To achieve adequate SCM contraction, the participants turned their head to the contralateral side. The cVEMP waveform was recorded in the SCM ipsilateral to the stimulus side. For oVEMP recording, active recording electrodes were placed approximately 1 cm below the midpoint of the lower orbital margin of both sides, and reference electrodes were placed approximately 1.5–2.0 cm below the active recording electrodes. The two electrodes on each side were placed symmetrically. When the stimulus was administered, the participants were instructed to gaze up at the top center of the head and to keep their eyes in a tense position approximately 30° upward. The oVEMP waveform was recorded below the eye contralateral to the stimulus side.

For GVS,^8^ we used 3-mA, 1-ms galvanic stimulation. The measured Electromyographic (EMG) activities were ampliﬁed and bandpass-ﬁltered (20–2000 Hz). The stimulation rate was 5 Hz, and the analysis time was 50 ms. The average of the responses to 50 stimuli was used. For artifact removal, we subtracted the average obtained without contraction of the SCM or extraocular muscle from the average obtained with contraction of the SCM or extraocular muscle. For ACS,^13^ the stimulus was a 500 Hz short tone burst at 123.5 dB SPL with a 1-ms rise/fall time and 2-ms peak duration. The stimulation rate was 5 Hz. The analysis time was 50 ms, and the average of 50 responses was taken for each run.

### Pure-tone audiometry

The average of Pure Tone Thresholds (PTAs) for air conduction and bone conduction were the averages of thresholds at 250-Hz, 500-Hz, 1-kHz, and 2-kHz frequencies. When the threshold could not be detected with the maximum sound output, the threshold was defined as 10 dB plus the maximum sound output.

### Data analysis

SPSS, version 18.0 for Windows (SPSS Inc., USA) was used for data analysis. Mean and Standard Deviation (SD) values are shown for the latencies of P1 and N1, VEMP amplitudes, and Amplitude Ratio (AR). The AR was calculated as |Ar − Al|/(Ar + Al) × 100%, where Ar is the amplitude in the right ear, Al is the amplitude in the left ear, and |Ar − Al| is the absolute value of (Ar − Al). A paired t-test was used to identify significant differences in the mean values for latency of P1 and N1, VEMP amplitudes, and AR between two groups. One-way analysis of variance (ANOVA) was used to identify significant differences in latency of P1 and N1, VEMP amplitudes, and AR among the clinical stages of MD. Bonferroni correction was used in multiple comparisons.

## Results

### Demographic characteristics of study participants

This retrospective study included 45 patients with MD and 164 normal individuals as the control group. The MD patients included 28 women and 17 men, with a mean age of 54.47 ± 12.11 years (range, 29–71 years). In the control group, 25 women and 15 men were matched based on sex and age, with a mean age of 53.40 ± 12.54 years (range, 29–71 years).

### Comparison of the provocation rates of VEMPs elicited by GVS and ACS

The rate of patients that can be elicited VEMP responses is defined as the provocation rate. The provocation rates to ACS and GVS were similar in both controls and MD patients. However, the MD patients presented a higher provocation rate of GVS-oVEMP, indicating the vestibular-ocular reflex of MD patients is more sensitive to GVS than to ACS (Table 1).

**Table 1:** Provocation rates of VEMPs by different stimuli.

	oVEMP			cVEMP		
	ACS	GVS	*p*	ACS	GVS	*p*
Control group (n = 164)	84.00%	93.29%	0.08	90.00%	95.12%	0.32
MD group (n = 45)	41.18%	86.67%	<0.001^a^ (0.00)	52.94%	95.56%	<0.001^a^ (0.00)
*p*		0.14			1.00	

a *p* < 0.05.

**Figure 1:**
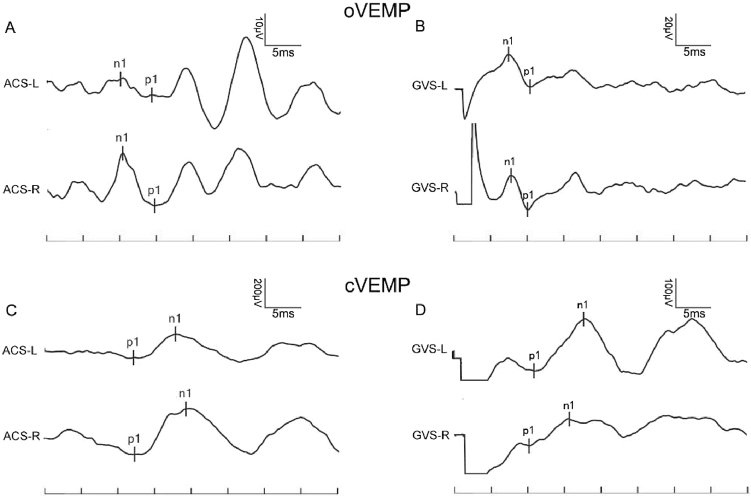
A 41-year-old woman with MD; the left ear was the affected ear. (A) ACS-oVEMP, (B) GVS-oVEMP, (C) ACS-cVEMP, and (D) GVS-cVEMP. VEMPs of both ears in this MD patient could be induced by ACS and GVS.

**Figure 2:**
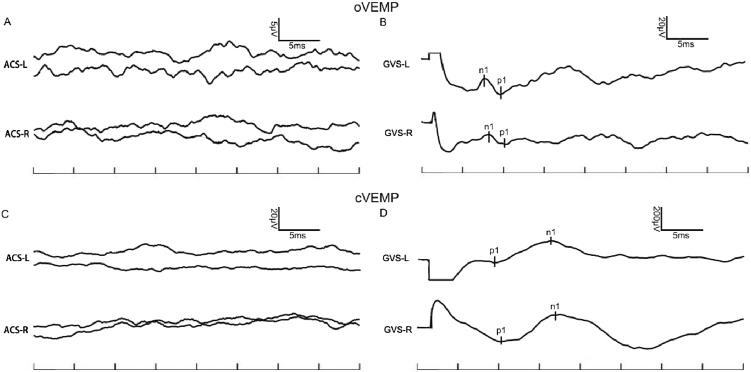
A 54-year-old woman with MD; the left ear was the affected ear. (A) ACS-oVEMP, (B) GVS-oVEMP, (C) ACS-cVEMP, and (D) GVS-cVEMP. VEMPs of both ears in this MD patient could be induced by GVS, but ACS-VEMPs were absent.

In the control group, the provocation rates of GVS-oVEMP and GVS-cVEMP were 93.29% and 95.12%, respectively, and these rates did not differ significantly from those in the MD patients (Table 1). Representative ACS-VEMP and GVS-VEMP waveforms are shown in Figure 1, Figure 2.

### Characteristic parameters of VEMPs induced by GVS and ACS in MD and control groups

To explore the response difference to ACS and GVS, we compared the latency and amplitude in controls and MD patients. In control group, the n1 and p1 latencies of GVS-oVEMP were 8.68 ± 1.42 ms and 11.89 ± 1.67 ms. In MD patients, the n1 and p1 latencies of GVS-oVEMP were 8.76 ± 2.05 ms and 11.06 ± 1.35 ms, respectively, and these values were significantly less than those of ACS-oVEMP. Similarly, the n1 and p1 latencies of GVS-cVEMP were also less than those of ACS-cVEMP. Thus, both controls and MD patients showed shorter latencies of VEMP evoked by GVS.

To further investigate whether GVS-VEMP in MD differs from that in normal individuals, we compared the characteristic parameters of GVS-VEMPs between the MD and control groups. Compared with the control group, the MD group showed no significant difference in n1 latency or p1 latency but exhibited significant reductions in the amplitude of both o- and c-VEMPs (Table 2).

**Table 2:** Parameters of VEMPs by different stimuli.

	n1 latency (ms)			p1 latency (ms)			Amplitude (μV)			AR (%)		
	Control	MD	*p*	Control	MD	*p*	Control	MD	*p*	Control	MD	*p*
oVEMP												
GVS	8.68 ± 1.42	8.76 ± 2.05	0.21	11.89 ± 1.67	11.38 ± 1.33	0.6	12.43 ± 5.50	7.96 ± 5.14	<0.001	23.34 ± 17.19	25.50 ± 18.12	0.02
ACS	11.76 ± 1.16	11.06 ± 1.35	0.14	15.76 ± 1.18	15.33 ± 1.28	0.19	9.08 ± 6.08	10.56 ± 7.53	0.51	20.23 ± 13.92	24.43 ± 21.75	0.07
*p*	0.00	<0.001^a^ (0.00)		0.00	<0.001^a^ (0.00)		0.95	0.34			0.42	
cVEMP												
GVS	18.21 ± 1.74	17.86 ± 1.87	0.67	11.20 ± 1.68	11.41 ± 1.59	0.16	127.66 ± 60.27	101.56 ± 52.18	0.02	23.63 ± 17.05	21.21 ± 12.46	0.43
ACS	20.26 ± 2.00	19.12 ± 1.63	0.004	12.94 ± 1.29	12.85 ± 1.92	0.04	107.84 ± 78.07	70.84 ± 70.22	0	19.87 ± 15.34	17.44 ± 16.57	0.30
*p*	0.00	0.003^a^ (0.003) (0.00)		0.00	<0.001^a^ (0.00)		0.93	0.05			0.55	

a P < 0.05.

We compared the characteristic parameters of ACS-VEMPs between the MD and control groups. Compared with the control group, the MD group showed no significant difference in n1 latency, p1 latency or amplitude of oVEMP, but exhibited a longer n1 and p1 latency of c-VEMP. Moreover, MD patients showed a lower amplitude of c-VEMP.

### Comparison of GVS-VEMPs according to the clinical stage of MD and correlations of GVS-VEMPs and auditory threshold

To explore whether GVS-VEMPs can reflect the stage of MD, we compared the characteristic parameters of GVS-VEMPs in patients with MD of different stages. The severity of MD was classified into four stages based on PTA:^5^ stage I, PTA < 25 dB; stage II, 26 < PTA < 40 dB; stage III, 41 < PTA < 70 dB; and stage IV, PTA > 70 dB. According to these criteria, the MD group in this study included 5 patients with stage I MD, 7 patients with stage II MD, 23 patients with stage III MD, and 7 patients with stage IV MD. Final auditory data were not available for 3 MD patients, and for this analysis, the control group consisted of 42 normal individuals matched to the 42 MD patients in terms of sex and age. We then compared the key parameters, including n1 latency, p1 latency, amplitude, and AR, of GVS-oVEMP and GVS-cVEMP among the stages of MD and observed no significant differences in any of the parameters among the four stages of MD for either o-VEMP or c-VEMP (Table 3). Furthermore, we found no correlations between the clinical stage of MD and the characteristic parameters (n1 latency, p1 latency, amplitude, and AR) of GVS-VEMPs (*p* > 0.05, bivariate correlation analysis).

**Table 3:** Comparison of GVS-VEMP parameters among stages I–IV of MD.

MD stage	oVEMP				cVEMP			
	n1 latency (ms)	p1 latency (ms)	Amplitude (μV)	AR	n1 latency (ms)	p1 latency (ms)	Amplitude (μV)	AR
I	8.43 ± 0.71	11.94 ± 0.60	10.05 ± 4.73	36.80 ± 20.99%	17.58 ± 1.79	11.79 ± 1.04	53.87 ± 27.44	25.30 ± 11.85%
II	8.40 ± 0.50	10.97 ± 0.49	6.92 ± 3.54	32.70 ± 29.83%	17.35 ± 2.19	11.37 ± 1.41	116.63 ± 58.63	22.05 ± 13.09%
III	9.00 ± 2.70	11.29 ± 1.63	7.73 ± 5.33	24.81 ± 15.43%	17.66 ± 1.51	11.05 ± 1.25	108.29 ± 56.84	19.78 ± 12.42%
IV	8.33 ± 0.83	11.47 ± 0.96	9.12 ± 6.49	17.78 ± 15.83%	18.26 ± 2.06	11.43 ± 2.21	88.42 ± 42.76	23.17 ± 16.78%
*p*	0.81	0.75	0.46	0.37	0.8	0.78	0.23	0.87

## Discussion

To explore the application of GVS-VEMP recording in the evaluation of MD, the current study examined the effectiveness of GVS for inducing VEMP and its ability to detect the localization and severity of MD. Among all MD patients in the present study, the provocation rates of ACS-oVEMP and ACS-cVEMP in the affected ears were 41.18% (14/34) and 52.94% (18/34), respectively. These rates are consistent with those reported among the 20 patients in the study by Murofushi et al. (60% and 55%, respectively).^5^ In the present study, the provocation rate of GVS-oVEMP and GVS-cVEMP in the affected ears were 86.67% (39/45) and 95.56% (43/45), respectively, and these rates seem higher than the corresponding rates (70% and 66%, respectively) reported by Wu et al.^1^ The high provocation rates of GVS-VEMP indicate the high sensitivity of this testing method.

Additionally, the results of the present study showed that the n1 and p1 latencies of both o-VEMP and c-VEMP elicited by GVS were shorter than those of o-VEMP and c-VEMP elicited by ACS. A likely explanation for these differences in that GVS directly triggers vestibular afferents and bypasses otolith organs. However, the change in the amplitude of GVS-VEMP was not confirmed. Chang et al.^14^ reported that the amplitude of GVS-cVEMP was smaller than that of ACS-cVEMP in both affected ears and unaffected ears. However, in another study 3 years later, the same group reported that this difference was non-significant.^1^ Our results are consistent with their most recent report, which showed no difference in amplitude. This indicates that the GVS fires as much vestibular neurons as ACS.

We found no statistically significant differences in the n1 and p1 latencies of GVS-oVEMP and GVS-cVEMP between the MD patients and the control group. The normal latencies observed indicate the conduction of the vestibular nerve is not affected in MD.

Compared with the control group, the MD patients showed a reduction in the amplitude of GVS-VEMP in the present study. In their 2017 study, Chang et al.^14^ also did not find this difference when they compared affected and nonaffected ears. This discrepancy could be caused by several factors, the first being the position of the recording electrodes. Placement of electrodes significantly above or below the middle of the SCM could lead to prolonged latencies and, at the extremes of the muscle, polarity inversions.^15^ A second potentially causative factor is individual variation. Different individuals’ skulls are shaped differently throughout development, which may cause differences in the stimulation of the otolith receptors.^16^

Jariengprasert et al. found that the endolymphatic hydrops were more extensive in the late stage of disease involving damage to the saccular structure and the inferior vestibular nerve, which might be a cause of the reduction in VEMP response, especially the VEMP amplitude.^17^ Our finding that fewer vestibular neurons fired in MD patients suggests that retrolabyrinthine degeneration exists in MD.

We further analyzed whether GVS-VEMP could be used to evaluate the severity of MD. Egami et al.^18^ suggested that with the progression of MD, labyrinthine lesions become increasingly serious. Previously, Young et al.^19^ recorded ACS-cVEMP in MD patients and calculated the Intramural Amplitude Difference (IAD) ratio (R − L/R + L), which is similar to the AR applied in the present study. They found that the IAD ratio was correlated with the stage of MD. Chang et al.^14^ also reported an association between MD stage and abnormal GVS-VEMP responses. In the present study, we did not detect any differences in GVS-VEMPs, or the characteristic parameters of GVS-VEMPs, among the four different MD stages. Perhaps potential variations in GVS-VEMPs with differing MD severity are too subtle to be detected, and thus, we performed correlation analysis, which also found no correlations between the clinical stage of MD and the characteristic parameters of GVS-VEMPs.

## Conclusions

GVS is as effective as ACS for inducing VEMPs, and even faster than ACS. GVS-VEMP recording can detect retrolabyrinthine degeneration in MD, and further research in more patients is needed to determine whether GVS-VEMP can reflect MD severity.

## Funding

This work was supported by 10.13039/501100001809National Natural Science Foundation of China (nº 81670945).

## Data statement

The data that support the findings of this study are available from the corresponding author upon reasonable request.

## Ethics committee statement

The present study was approved by the Ethics Committee of the Second Affiliated Hospital of Xi’an Jiaotong University (2016205). Informed consent was obtained from all study participants.

## Data availability

Data will be made available on request.

## Conflicts of interest

The authors declare no conflicts of interest.
